# Facets of shame and their impact on quality of life in patients with atopic dermatitis and psoriasis

**DOI:** 10.1038/s41598-025-98353-w

**Published:** 2025-04-21

**Authors:** Carsten Spitzer, Laura Lübke, Clara Wülfing, Katharina Meier, Georgios Kokolakis, Dennis Niebel, Caroline Mann, Ante Karoglan, Steffen Emmert, Alexander Thiem

**Affiliations:** 1 Department of Psychosomatic Medicine and Psychotherapy, University Medical Center Rostock, Gehlsheimer Str. 20, 18147 Rostock, Germany; 2 Clinic and Policlinic for Dermatology and Venereology, University Medical Center Rostock, Rostock, Germany; 3 Department of Dermatology, Venereology and Allergology, Charité-Universitätsmedizin Berlin, Berlin, Germany; 4 Department of Dermatology, University Hospital Regensburg, Regensburg, Germany; 5 Department of Dermatology, University Medical Center of the Johannes Gutenberg University, Mainz, Germany; 6 Dermatology Practice, Berlin, Germany

**Keywords:** Atopic dermatitis, Psoriasis, Shame, Quality of life, Cross-sectional study, Skin diseases, Human behaviour

## Abstract

Shame is a complex emotion with different facets. Skin shame is a specific aspect of body shame, which involves the skin perceived as inferior or flawed. Its role in atopic dermatitis (AD) and psoriasis is not well investigated. This explorative study pursued three objectives: First, the comparative analysis of shame and its facets in AD and psoriasis. Second, analysis of the association of skin shame with other shame facets, depression, and anxiety. Third, exploration of the unique impact of shame and its different facets on quality of life (QoL) in AD and psoriasis patients. This cross-sectional online survey encompassing German-speaking patients included several self-report measures on skin and general shame, depression, and anxiety as well as the Dermatology Life Quality Index (DLQI), Patient-Oriented Eczema Measure (POEM), and Psoriasis Symptoms and Signs Diary (PSSD). Data from 413 adult participants with AD (*N* = 162) or psoriasis (*N* = 251) were analyzed. There were no significant differences in skin or general shame, depression, or anxiety between those with AD or psoriasis. Skin shame as well as other aspects of shame were associated with younger age, female sex, depression, anxiety, and QoL. Analysis of AD and psoriasis subsamples revealed significant correlations of disease severity with skin shame, depression, anxiety, and DLQI. Hierarchical linear regression analyses indicated that skin shame was the second most important determinant of QoL after self-assessed disease severity. Systematic consideration of shame in AD and psoriasis is necessary in order to effectively reduce disease burden and enhance QoL.

**Supplementary Information:**

The online version contains supplementary material available at 10.1038/s41598-025-98353-w.

## Introduction

Shame is a complex emotion characterized by the desire to be “unseen” and the perception of being worthless, inferior, and flawed. It is considered “one of the most powerful, painful, and potentially destructive experiences known to humans” because it concerns the entire self^1^. Shame is elicited during real or imagined social interactions, and plays a crucial role in psychosocial functioning^2,3^.

Shame has been conceptualized as a multifaceted construct^4^. For example, a facet labeled cognitive shame arises after trespassing on one’s personal or moral values. Similarly, body or bodily shame is elicited if one fails to meet her or his standards according to the individual or culturally accepted body ideal^4^. General shame can be considered a severe threat to the self and is closely associated with distress and psychopathology in general^5^ and in particular with depression^6^, and anxiety^3,7,8^.

One specific aspect of body shame, i.e. experiencing one’s own body as unattractive, undesirable, or not meeting (internalized) societal ideals of attractiveness, refers to skin shame, i.e. the skin is perceived as inferior and flawed^9–11^. Thus, individuals with dermatological diseases, particularly those with marked alterations of the skin and affecting body parts usually visible in social interactions, may be especially vulnerable to shame.

Despite the theoretically convincing link between skin disease and shame, the research interest in dermatology has been limited and is just beginning to emerge^12,13^. Conditions seen in dermatology that have been studied with respect to shame include body-focused repetitive behaviors^14,15^, acne^16^, severe burns^17^, excess skin due to extreme weight loss^18^, and eczematous skin diseases such as atopic or contact dermatitis^19^.

In another, cross-sectional study, 171 patients with psoriasis reported significantly higher skin shame compared to sex-matched skin-healthy controls^20^. Similar findings were observed in 44 psoriasis patients who rated their skin shame significantly higher than a control group of 88 age- and gender-matched individuals without any skin problems^13^. Of note, bodily, and cognitive shame did not differ between the two groups. Among psoriasis patients, skin shame was associated with the self-rated disease burden as well as the health-related quality of life (QoL), but not with psychological distress, general shame, or expert-rated disease severity^13^. In contrast, there were significant correlations between skin shame, general shame, and psychological distress in another study^10^. Likewise, a prospective observational study indicated significantly higher levels of skin and general shame among 201 consecutive dermatological outpatients with a variety of diagnoses compared to individuals free of skin diseases^12^. Within the patient group, those affected by psoriasis (*n* = 49), atopic dermatitis (AD; *n* = 22) or other inflammatory skin diseases (*n* = 35) exhibited the highest levels of skin shame but did not differ in other shame dimensions from patients with tumors, infections or allergic conditions^12^. Skin shame, but no other shame facet was associated with disease duration and lesion visibility^12^.

In sum, few studies have demonstrated the importance of shame in dermatological conditions, particularly in those with marked and visible skin lesions, e.g. psoriasis or AD. However, prior research has yielded inconclusive findings regarding the association between skin shame, other shame dimensions, and psychopathology. Specifically, the potential correlations between skin shame, depression, and anxiety have not yet been thoroughly investigated. Of note, these two conditions are frequent in psoriasis and AD, respectively^21,22^. Moreover, although skin shame was related to QoL in bivariate analyses, its unique impact on QoL when considering other important determinants^23–26^, such as depression, anxiety, and skin disease-related factors (i.e. severity and onset), remains unknown. Finally, the sample sizes studied in previous investigations were small in some instances.

Thus, this explorative study aimed to deepen our understanding of shame in AD and psoriasis. The specific research questions were: (i) Do patients with AD and psoriasis, respectively, differ concerning shame?; (ii) Is skin shame associated with other facets of shame, depression, and anxiety?; (iii) How does shame affect QoL, and which shame dimension uniquely impacts QoL, independent of other relevant factors?

## Patients and methods

### Study design and procedure

Adult participants with either psoriasis or AD were recruited through a German online survey posted on various websites thought to be visited by affected people (i.e., websites of the involved departments and offices, support groups, self-help resources) and in several social media channels. Furthermore, the study was made public through a poster notice in the dermatology departments and cooperating offices. The survey took place between July 25th, 2023, and April 29th, 2024. All participants were provided informed consent during the study, and then completed an assessment battery. Anonymity was guaranteed and there was no monetary or other compensation for completing the full survey. The study was approved by the Ethics Committee of the University Medical Center Rostock (approval number A 2023-0051) and conformed to the principles of the Declaration of Helsinki.

### Self-report measures

Before completing the following questionnaires, participants were asked to provide sociodemographic and skin disease-related information, e.g. age at symptom onset to determine disease duration.

The *Shame Assessment for Multifarious Expressions of Shame (SHAME)* allows to measure shame-proneness with 21 items describing potentially shameful scenarios^4^. On a six-point Likert scale, participants indicate how much they anticipate feeling ashamed in response to these scenarios (0 = not at all, 5 = very strong). It consists of the three scales bodily (relating to the body ideal), cognitive (referring to the person´s moral standards), and existential shame (describing an enduring feeling of shame comprising someone’s person as a whole); additionally, a total score indicating general shame can be calculated. The SHAME showed adequate reliability and factorial validity^4^. Furthermore, internal consistency was good across different patient samples with Cronbach´s α ranging from 0.82 to 0.94^4^.

The *Skin Shame Scale (SSS-24)* was used to capture an individual’s skin-related shame in the prior week^10^. Referring to models of shame in the context of dermatological disease and body awareness, the scale comprises 24 items, which are to be rated on a five-point scale ranging from 1 to 5 with higher scores indicating a higher degree of skin shame^11^. The German translation was psychometrically evaluated in 488 skin-healthy individuals and 339 dermatological patients. Factor analyses revealed the unidimensional structure of the SSS-24; it correlated significantly with the SHAME questionnaire and self-rated psychological distress. Internal consistency was excellent (α = 0.95)^10^.

The *Patient Health Questionnaire-4 (PHQ-4)* is an ultra-brief screening instrument for depression and anxiety^27^. Two items cover the core symptoms of depression (PHQ-2), and another two items capture the core symptoms of anxiety (GAD-2) over the past two weeks. The total PHQ-4 score represents an overall measure of symptom burden. Each item is scored on a four-point Likert scale ranging from 0 (not at all) to 3 (nearly every day). Higher scores denote greater levels of depression and anxiety, respectively. There is ample evidence for the reliability and validity of the PHQ-4 and its subscales^28^.

The *Dermatology Life Quality Index (DLQI)* is a well-established, generic measure of health-related QoL in the last week among patients with skin diseases^29^. Its ten items relate to the effect of skin problems on six aspects of life-labeled individual domains: symptoms and feelings including shame, daily activities, leisure, work and school performance, personal relationships, and treatment. The items are rated on four-point Likert scales from 0 (not at all) to 3 (very much), generating sum scores with higher scores indicating a lower QoL. Reliability and validity have been established in numerous studies^30^. In addition to the sum score, we also calculated an alternative DLQI score (DLQI_[−item 2]_) which disregards the one item explicitly relating to shame, thus reducing a possible bias due to shared variance.

The *Patient-Oriented Eczema Measure (POEM)* is the preferred self-report instrument for the assessment of AD symptom severity in clinical trials and observational studies^31–33^. It is composed of seven questions evaluating dryness, itching, flaking, cracking, bleeding, and weeping of the skin as well as sleep disturbance in the past week. Each item can be rated on a five-point scale ranging from 0 (no days) to 4 (every day). The POEM has extensively been validated with adequate reliability and validity^31,32^.

The *Psoriasis Symptoms and Signs Diary (PSSD)* is an internationally well-established and validated measure assessing psoriasis severity^34,35^. Referring to the past week, patients are asked to rate five symptoms and six signs on an 11-point scale from 0 (absent) to 10 (worst imaginable). In addition to mean symptom and sign scores, a total score ranging from 0 to 100 is calculated with higher scores indicating greater self-perceived disease severity. The PSSD has strong psychometric properties^35,36^.

### Data analysis

In addition to descriptive statistics, several between-group comparisons between patients with AD and psoriasis were performed. For categorical variables, the χ^2^-test was used. The t-test was applied for continuous variables; the magnitude of the difference was estimated by effect sizes (Cohen’s d). Bivariate associations were evaluated by correlation coefficients (Pearson’s r); coefficients of |r| < 0.3 are considered weak, values between 0.3 and < 0.5 indicate a moderate correlation, and values between ≥ 0.5 suggest a strong correlation. To determine the differential effect of sociodemographic characteristics, skin disease-related features, psychopathology, as well as skin shame and other facets of shame on QoL, hierarchical linear regression analyses were run with DLQI scores as the dependent variable and for each of the subsamples (i.e. the AD and psoriasis patients, respectively) separately. Age, sex, disease severity, and duration were entered in the 1st block; PHQ-2 and GAD-2 scores were considered in the 2nd block; the SSS-24 and SHAME subscale scores were entered in the 3rd block. The adjusted explained variance (Adj. R^2^), the increase in explained variance (ΔR^2^) when adding further variables in subsequent blocks, the unstandardized regression coefficient B, its corresponding standard error (SE), and the standardized regression coefficient β are reported. The same analyses were repeated with DLQI_(−item 2)_ as the dependent variable to account for possible overlaps between shame and QoL. There was no evidence of collinearity as indicated by variance inflation factors, which were below 3 in all cases. Significance level was set at *p* < 0.05. All analyses were computed using the ‘Statistical Package for the Social Sciences‘ (SPSS, version 27.0, IBM, Armonk, NY, USA).

## Results

A total of 467 individuals took part in the survey, but 54 (11.5%) had to be excluded due to missing data. Among the final study sample (*N* = 413 participants), there were 267 women (64.6%) and 146 men (35.4%) with a mean age of 43.0 years (SD = 15.7; range: 18–85 years). Of those, 162 (39.2%) suffered from AD and 251 (60.8%) from psoriasis, respectively. A more detailed account of the sociodemographic and skin disease-related clinical characteristics of the study population and the two subsamples is presented in Table 1.

**Table 1:** Sociodemographic and clinical characteristics of the study population.

	Study sample	AD patients	Psoriasis patients	Statistics	
	(*N* = 413)	(*N* = 162)	(*N* = 251)	T/ χ^2^	p
**Age** (years; M ± SD; range)	43.0 ± 15.7 (18–85)	37.2 ± 14.5 (18–80)	46.7 ± 15.3 (18–85)	-6.28	< 0.001
**Sex**				4.69	0.030
Women	267 (64,6%)	115 (71.0%)	152 (60.6%)		
Men	146 (35.4%)	47 (29.0%)	99 (39.4%)		
**Marital status**				12.63	0.002
Single/ unmarried	175 (42.7%)	85 (52.8%)	99 (36.1%)		
Married/ steady partner	199 (48.5%)	61 (37.9%)	138 (55.4%)		
Separated/ widowed	36 (8.8%)	15 (9.3%)	21 (8.4%)		
Education*				10.69	0.005
Lower secondary education (level 2)	178 (43.1%)	54 (33.3%)	124 (49.4%)		
Upper secondary education (level 3)	222 (53.8%)	103 (63.6%)	119 (47.4%)		
Other^#^	13 (3.1%)	5 (3.1%)	8 (3.2%)		
**Disease duration** (years; M ± SD)	24.2 ± 16.3	27.0 ± 14.8	22.3 ± 17.0	3.00	0.003
**Disease severity**					
POEM, 0–28 (M ± SD)		13.4 ± 6.9			
PSSD, 0-100 (M ± SD)			25.6 ± 23.6		
PSSD symptoms			12.7 ± 11.9		
PSSD signs			20.4 ± 15.3		
**DLQI**, 0–30 (M ± SD)	7.35 ± 6.61	8.59 ± 6.70	6.55 ± 6.44	3.09	0.002
**DLQI**_**(−item 2)**_ (M ± SD)	6.39 ± 5.86	7.45 ± 5.93	5.70 ± 5.73	2.99	0.003

AD, atopic dermatitis; M, mean; SD, standard deviation; T, t-test statistic; χ^2^, chi-square statistic; p, p-value; POEM, Patient-Oriented Eczema Measure; PSSD, Psoriasis Symptoms and Signs Diary; DLQI, Dermatology Life Quality Index; DLQI_(−item 2)_, Dermatology Life Quality Index without its Item 2.

* according to the International Standard Classification of Education (ISCE); corresponding to the German school system, education was categorized into ≤ 10 years which aligns with level 2 of the ISCE, and ≥ 11 years (level 3).

^#^ this category includes ‘still in school’, ‘no graduation’, ‘ special school’, and ‘other’.

There were no significant differences in skin shame, SHAME total score and its subscales, depression, and anxiety between the two subsamples (Table 2).

**Table 2:** Psychosocial characteristics in patients with chronic inflammatory skin diseases.

	Study sample	AD patients	Psoriasis patients			
	(*N* = 413)	(*N* = 162)	(*N* = 251)	T	p	d
**SSS-24**, 24–120	66.1 ± 18.0	67.9 ± 17.2	65.0 ± 18.4	1.68	0.093	0.17
**SHAME**, 0–5	2.14 ± 0.77	2.18 ± 0.76	2.11 ± 0.77	0.87	0.385	0.09
Bodily shame, 0–5	2.09 ± 1.12	2.17 ± 1.11	2.04 ± 1.13	1.17	0.241	0.12
Cognitive shame, 0–5	3.41 ± 1.03	3.48 ± 1.01	3.37 ± 1.04	1.03	0.304	0.10
Existential shame, 0–5	0.91 ± 0.77	0.88 ± 0.71	0.92 ± 0.81	-0.51	0.609	− 0.05
**PHQ-4**, 0–6	4.43 ± 3.24	4.73 ± 3.26	4.23 ± 3.21	1.56	0.120	0.16
PHQ-2, 0–6	2.11 ± 1.67	2.29 ± 1.68	2.00 ± 1.66	1.75	0.081	0.18
GAD-2, 0–6	2.31 ± 1.83	2.44 ± 1.87	2.23 ± 1.80	1.16	0.247	0.12

AD, atopic dermatitis; T, t-test statistic; p, p-value; d, Cohen’s d effect size; SSS-24, Skin Shame Scale; SHAME, Shame Assessment for Multifarious Expressions of Shame; PHQ-4, Patient Health Questionnaire-4; PHQ-2, depression items of the Patient Health Questionnaire-4; GAD-2, anxiety items of the Patient Health Questionnaire-4.

Table 3 illustrates the intercorrelations of the variables of interest in the entire study population. Younger age and female sex were significantly related to all shame facets except existential shame, psychopathology, and QoL. Skin shame was strongly associated with QoL explaining 49% of shared variance. Regarding other aspects of shame, skin shame was most closely linked to bodily shame (*r* = 0.41), and least closely, but still significant to cognitive shame (*r* = 0.23). Moreover, there were significant correlations between skin shame, depression, and anxiety. Depression, anxiety, and QoL were significantly interrelated, too.

**Table 3:** Intercorrelations (Pearson r) between the variables of interest.

	Age	Sex	SSS-24	SHAME	Bodily	Cognitive	Existential	PHQ-2	GAD-2
**Sex**	0.18***	-							
**SSS-24**	− 0.14**	− 0.30***	-						
**SHAME**	− 0.18***	− 0.31***	0.41***	-					
**Bodily**	− 0.22***	− 0.39**	0.41***	0.89***	-				
**Cognitive**	− 0.14**	− 0.26***	0.23***	0.79***	0.57***	-			
**Existential**	− 0.03	− 0.02	0.31***	0.63***	0.44***	0.18***	-		
**PHQ-2**	− 0.15***	− 0.20***	0.56***	0.35***	0.30***	0.22***	0.31***	-	
**GAD-2**	− 0.17***	− 0.28***	0.53***	0.42***	0.37***	0.25***	0.39***	0.71***	-
**DLQI**	− 0.12**	− 0.24***	0.70***	0.25***	0.23***	0.13**	0.23***	0.52***	0.48***
**DLQI**_**(−item 2)**_	− 0.11*	− 0.23***	0.68***	0.23***	0.21**	0.12*	0.22***	0.51***	0.47***

SSS-24, Skin Shame Scale; SHAME, Shame Assessment for Multifarious Expressions of Shame; PHQ-2, depression items of the Patient Health Questionnaire-4; GAD-2, anxiety items of the Patient Health Questionnaire-4; DLQI, Dermatology Life Quality Index; DLQI_(−item 2)_, Dermatology Life Quality Index without its Item 2.

*p < 0.05, **p < 0.01, ***p < 0.001.

Analyzing the two subsamples separately also allowed disease duration and disease severity to be included (Supplementary Table S1). In AD patients, disease severity was closely linked to skin shame, depression, anxiety, and QoL, but not to other facets of shame. Disease duration did not correlate with any of the aforementioned variables. Almost identical results were obtained among psoriasis patients; additionally, disease severity was significantly associated with general shame and all subscales except for cognitive shame. Disease duration had significant negative correlations with bodily shame, depression, and anxiety.

To account for the multifactorial determinants of QoL and to determine the unique effect of shame, hierarchical linear regression analyses were performed for the AD and psoriasis patients separately (Table 4).

**Table 4:** Associations between quality of life (as measured by the DLQI [dependent variable]) and sociodemographic characteristics, skin disease-related features, depression, anxiety, and shame dimensions (hierarchical linear regression).

	AD patients			Psoriasis patients		
	Adj. *R*^2^	ΔR^2^	*p*	Adj. *R*^2^	ΔR^2^	*p*
**Block 1**	0.53	0.54	< 0.001	0.52	0.53	< 0.001
**Block 2**	0.59	0.07	< 0.001	0.57	0.04	< 0.001
**Block 3**	0.64	0.05	< 0.001	0.66	0.10	< 0.001

Variables	B ± SE	β	p	B ± SE	Β	p
**Disease severity** ^#^	0.44 ± 0.06	0.46	< 0.001	0.12 ± 0.01	0.45	< 0.001
**Disease duration**	− 0.04 ± 0.02	− 0.08	0.136	− 0.01 ± 0.02	− 0.04	0.412
**Age**	0.00 ± 0.03	0.01	0.864	0.01 ± 0.02	0.03	0.587
**Sex**	− 0.33 ± 0.79	− 0.02	0.674	− 0.70 ± 0.56	− 0.05	0.219
**Depression** (PHQ-2)	0.17 ± 0.28	0.05	0.541	0.42 ± 0.22	0.11	0.057
**Anxiety** (GAD-2)	0.40 ± 0.26	0.11	0.129	− 0.13 ± 0.21	− 0.04	0.526
**Skin Shame** (SSS-24)	0.13 ± 0.03	0.34	< 0.001	0.15 ± 0.02	0.43	< 0.001
**Bodily Shame**	− 0.12 ± 0.43	− 0.02	0.776	− 0.65 ± 0.31	− 0.11	0.036
**Cognitive Shame**	0.01 ± 0.41	0.00	0.977	0.17 ± 0.28	0.03	0.552
**Existential Shame**	− 0.02 ± 0.53	− 0.00	0.973	0.57 ± 0.37	0.07	0.112

Adj. R^2^, explained variance; ΔR^2^, increase in explained variance; B ± SE, unstandardized regression coefficient with standard error; β, standardized regression coefficient.

Block 1: Inclusion of disease severity, disease duration, age, and sex.

Block 2: Additional inclusion of depression (PHQ-2) and anxiety (GAD-2).

Block 3: Additional inclusion of skin shame (SSS-24) and other facets of shame (SHAME subscales).

^#^ For AD patients, the POEM was used; among psoriasis patients, the PSSD was applied.

In both subsamples, the additional inclusion of shame dimensions into the regression equation yielded a significant increase in explained variance over and above sociodemographic features, disease-related factors as well as depression and anxiety, respectively. Of note, among all factors included in the models, skin shame emerged as the second most important determinant of QoL after disease severity, both in AD and psoriasis patients. Skin shame was a significant predictor on QoL in psoriasis patients, contributing to a notable increase in explained variance when entered into the regression equation and demonstrating a strong β coefficient. Similarly, in AD patients, skin shame was also a significant predictor of QoL, although the magnitude of its contribution was different within this subsample. Rerunning these analyses with *DLQI*_*(−item 2)*_ as dependent variable yielded consistent findings in both subsamples (Supplementary Table S2).

## Discussion

Given the limited, but increasingly growing scientific and clinical interest in the complex emotion of shame in dermatology^12,13,16,19,20^, this study aimed at further exploring its relevance and correlates in AD and psoriasis^37,38^. Our findings suggest that skin shame is a major concern in patients with AD and psoriasis, because of its tight association with QoL, depression, anxiety, and self-rated disease severity. Moreover, skin shame emerged as the second most important predictor of QoL after disease severity, i.e. it was more strongly associated with QoL than depression and anxiety.

While skin-healthy individuals were reported to typically endorse scores of about 44 on the *Skin Shame Scale* (*SSS-24*)^10,13^, dermatological patients’ mean score was significantly higher with 63.2 ± 21.8^10,12^. The large standard deviation of the *SSS-24* score seen in patients with skin diseases indicates that skin shame is of minor importance in some conditions, but plays an important role in others. Indeed, patients with psoriasis, other inflammatory skin diseases, and eczema had the highest *SSS-24* scores while those affected by allergic diseases or tumors had scores only slightly above those reported for skin-healthy persons^12^. In this study, AD and psoriasis patients did not differ in their degree of skin shame and had almost identical *SSS-24* scores compared to the ones found for psoriasis and eczema patients in prior research^12,13^. Consistently, another study found that skin-related shame as assessed by the Touch-Shame-Disgust questionnaire was significantly higher in psoriasis patients compared to a healthy control group^20^.

From a theoretical point of view, skin shame can be conceptualized as a specific aspect of body shame^9^, which in turn is considered one facet of the complex and multidimensional emotion of shame^4^. Correspondingly, the correlation of the *SSS-24* was highest for the subscale bodily shame of the *SHAME*, and lowest for the subscale cognitive shame in this study. A very similar relation between the *SSS-24* and the *SHAME* including an almost identical magnitude of the correlation coefficients was reported by another publication^10^.

While shame is linked to psychopathology or general psychological distress in non-clinical and clinical populations^5,13,39,40^, its association with anxiety and depression in dermatological patients has not yet been explored. This is surprising for two reasons. First, both depression and anxiety are frequent in this population, particularly in those with AD and psoriasis^21,22^. Second, there is meta-analytic evidence that shame is related to anxiety and depressive symptoms with medium effect sizes^6,7^. Consistently, the correlations of the *PHQ-2* and the *GAD-2*, respectively, with the *SSS-24* in this study, indicated medium effect sizes. In contrast, the correlations with other dimensions of shame were lower ranging from *r* = 0.22 (*PHQ-2* with the cognitive subscale of the *SHAME*) to *r* = 0.42 (*GAD-2* with *SHAME* total score). Thus, it could be concluded that skin shame is more important than other facets of shame concerning depression and anxiety in AD and psoriasis patients.

Controlling for disease severity as a major determinant of QoL^23–26^, skin shame significantly predicted the *DLQI* scores in both the AD and psoriasis subsamples, but anxiety and depression did not (with the exception that depression just reached significance in psoriasis patients). Notably, this interesting result came as a surprise because it puts into perspective the importance of depression and anxiety which have been identified as important determinants of QoL in prior research^24,41–48^.

Pending replication, our findings hold important clinical implications. Assuming that skin shame is at least as relevant as depression and anxiety in both AD and psoriasis patients and substantially impacts their QoL, addressing this painful emotion through systematic screening within routine assessment might be beneficial. Moreover, psychosocial interventions to reduce shame may help to increase QoL^49^. In other medical conditions, shame represents a barrier to seeking treatment and has been related to poor treatment adherence, unfavorable outcomes, and premature therapy termination^49–51^. Future studies are warranted to clarify whether this also holds for AD and psoriasis.

Nevertheless, some methodological limitations merit discussion. First, because patients were recruited online, self-selection bias cannot be ruled out and the generalizability of the reported findings remains unclear. However, we strived to address this issue by recruiting study participants in dermatology departments and offices as well as by involving support groups. Second, skin disease severity was self-rated by patients and was not based on expert assessments. Despite this limitation, patient-reported outcomes are increasingly important in clinical decision-making in AD and psoriasis^52,53^. Third, the cross-sectional design precludes any temporal or causal inferences, particularly regarding the correlations between skin shame, depression, anxiety, and QoL. For example, skin shame might result in depression; conversely, depression may increase shame-proneness. Most likely, the relations are bidirectional. Moreover, because lesion location was not assessed, its relation to skin shame and its impact on QoL could not be determined. Finally, mean DLQI values were in the moderate range and below the cut-off indicating severe effects.

Notwithstanding these caveats, this study in concert with prior findings^12,13,20^ suggests that skin shame is an important issue in AD and psoriasis. Shame contributes to and maintains social as well as self-stigmatization^54,55^, which in turn may additionally impair QoL and psychosocial functioning in many areas of life among affected individuals^56,57^. Thus, adequately addressing shame might help to alleviate AD and psoriasis patients’ disease burden.

## Electronic supplementary material

Below is the link to the electronic supplementary material.

Supplementary Material 1

## Data Availability

The data that support the findings of this study are not openly available due to reasons of sensitivity and are available from the corresponding author upon reasonable request. Data are located in controlled access data storage at the University Medical Center Rostock.
