# Supplementary material for: Facets of shame and their impact on quality of life in patients with atopic dermatitis and psoriasis
Source: Sci Rep. 2025 Apr 21;15:13753. doi: 10.1038/s41598-025-98353-w (PMC12012170; doi:10.1038/s41598-025-98353-w)
Supplement: Supplementary file 1 — Supplementary Material 1 [file 41598_2025_98353_MOESM1_ESM.docx]

**Supporting Information**

**Table S1:** Intercorrelations (Pearson r) between the variables of interest in patients with AD (lower left) and psoriasis (upper right).

|  |  | **1.** | **2.** | **3.** | **4.** | **5.** | **6.** | **7.** | **8.** | **9.** | **10.** | **11.** | **12.** | **13.** |
| --- | --- | --- | --- | --- | --- | --- | --- | --- | --- | --- | --- | --- | --- | --- |
| **1** | **Age** | - | .13* | -.07 | -.15* | -.16* | -.10 | -.07 | -.14* | -.16* | -.06 | -.05 | .49*** | -.07 |
| **2** | **Sex** | .20* | - | -.31*** | -.29*** | -.37*** | -.23*** | -.03 | -.20*** | -.30*** | -.28*** | -.27*** | .15* | -.26*** |
| **3** | **SSS-24** | -.21** | -.26*** | - | .38*** | .39*** | .20** | .31*** | .53*** | .49*** | .72*** | .70*** | -.08 | .56*** |
| **4** | **SHAME** | -.23** | -.34*** | .44*** | - | .90*** | .77*** | .63*** | .38*** | .43*** | .25*** | .24*** | -.12 | .19** |
| **5** | **Bodily** | -.30*** | -.41*** | .43*** | .89*** | - | .55*** | .46*** | .33*** | .38*** | .23*** | .21** | -.13* | .20** |
| **6** | **Cognitive** | -.20** | -.31*** | .28*** | .82*** | .60*** | - | .14* | .21*** | .20*** | .11 | .11 | -.07 | .06 |
| **7** | **Existential** | .04 | .00 | .32*** | .64*** | .43*** | .26*** | - | .36*** | .45*** | .26*** | .25*** | -.09 | .18** |
| **8** | **PHQ-2** | -.12 | -.18* | .60*** | .30*** | .24*** | .23** | .25*** | - | .72*** | .50*** | .49*** | -.16** | .41*** |
| **9** | **GAD-2** | -.18* | -.23** | .60*** | .40*** | .34*** | .32*** | .30*** | .69*** | - | .45*** | .44*** | -.15* | .42*** |
| **10** | **DLQI** | -.13 | -.15* | .68*** | .23** | .21** | .14 | .20* | .54*** | .52*** | -* | 1.00*** | -.11 | .72*** |
| **11** | **DLQI_(-item 2)_** | -.11 | -.14 | .64*** | .21** | .19* | .13 | .19* | .53*** | .50*** | .99*** | - | -.10 | .71*** |
| **12** | **Dis. duration** | .32*** | -.05 | -.05 | .05 | -.04 | .12 | .07 | -.04 | -.01 | -.10 | -.10 | - | -.08 |
| **13** | **Dis. severity^#^** | -.13 | -.12 | .56*** | .13 | .11 | .07 | .14 | .50*** | .41*** | .72*** | .71*** | -.01 | - |

SSS-24, Skin Shame Scale; SHAME, Shame Assessment for Multifarious Expressions of Shame; PHQ-2, depression items of the Patient Health Questionnaire-4; GAD-2, anxiety items of the Patient Health Questionnaire-4; DLQI, Dermatology Life Quality Index; DLQI_(-item 2)_, Dermatology Life Quality Index without its Item 2; Dis, Disease

*p < 0.05, **p < 0.01, ***p < 0.001,

^#^ For AD patients, the POEM was used; among psoriais patients, the PSSD was applied

**Table S2:** Associations between quality of life (as measured by the DLQI_(-item 2)_ [dependent variable]) and sociodemographic characteristics, skin disease-related features, depression, anxiety, and shame dimensions (hierarchical linear regression).

|  | **AD** | | |  | **Psoriasis** | | |
| --- | --- | --- | --- | --- | --- | --- | --- |
|  | **Adj. R^2^** | **ΔR^2^** | **p** |  | **Adj. R^2^** | **ΔR^2^** | **p** |
| **Block 1** | .51 | .53 | <.001 |  | .51 | .52 | <.001 |
| **Block 2** | .57 | .06 | <.001 |  | .55 | .04 | <.001 |
| **Block 3** | .60 | .04 | .005 |  | .64 | .09 | <.001 |
| **Variables** | **B ± SE** | **β** | **p** |  | **B ± SE** | **β** | **p** |
| **Disease severity**^#^ | .39 ± .05 | .47 | <.001 |  | .11 ± .01 | .44 | <.001 |
| **Disease duration** | -.03 ± .02 | -.08 | .144 |  | -.01 ± .02 | -.04 | .429 |
| **Age** | .01 ± .02 | .02 | .767 |  | .01 ± .02 | .03 | .502 |
| **Sex** | -.33 ± .73 | -.03 | .650 |  | -.64 ± .52 | -.06 | .215 |
| **Depression** (PHQ-2) | .21 ± .26 | .06 | .422 |  | .41 ± .20 | .12 | .046 |
| **Anxiety** (GAD-2) | .35 ± .24 | .11 | .150 |  | -.14 ± .19 | -.04 | .471 |
| **Skin Shame** (SSS-24) | .10 ± .03 | .30 | <.001 |  | .13 ± .02 | .41 | <.001 |
| **Bodily Shame** | -.16 ± .39 | -.03 | .691 |  | -.69 ± .28 | -.14 | .016 |
| **Cognitive Shame** | .02 ± .37 | .00 | .959 |  | .20 ± .26 | .04 | .431 |
| **Existential Shame** | .05 ± .49 | .01 | .911 |  | .55 ± .34 | .08 | .105 |

Adj. R^2^, explained variance; ΔR^2^, increase in explained variance; B ± SE, unstandardized regression coefficient with standard error; β, standardized regression coefficient

Block 1: Inclusion of disease severity, disease duration, age, and sex

Block 2: Additional inclusion of depression (PHQ-2) and anxiety (GAD-2)

Block 3: Additional inclusion of skin shame (SSS-24) and other facets of shame (SHAME subscales)
